# 多原发肺癌的诊疗

**DOI:** 10.3779/j.issn.1009-3419.2018.03.12

**Published:** 2018-03-20

**Authors:** 黎杰 谭, 俊 尹

**Affiliations:** 复旦大学附属中山医院胸外科 Department of Toracic Surgery, Zhongshan Hospital Afliated to Fudan University

韩连奎等的关于同时性多原发肺癌的研究，总结了31例同时性多原发肺癌的治疗体会，并归纳出如下经验：(1)薄层高分辨计算机断层扫描(computed tomography, CT)是术前诊断同时性多原发肺癌的最佳方法；(2)病灶位于同侧者可同期行手术治疗，手术方式以胸腔镜下肺叶+亚肺叶切除为主；病灶位于双侧者，可分期手术，时间间隔为3个月-4个月；(3)胸腔镜下主病灶的肺叶切除+次要病灶的亚肺叶切除是最常用的术式。本文结合该文章，就多原发肺癌的诊疗现状作简单概述和评论。

## 1 诊断

自从Martini和Melamed于1975年首次提出同时性多原发肺癌(synchronous multiple primary lung cancers, sMPLC)及异时性多原发肺癌(metachronous multiple primary lung cancers, mMPLC)概念，将其从肿瘤肺内转移中划分出来^[1]^，随着CT等诊断技术的提高，MPLC的检出率逐年增加。研究显示，在进行非小细胞肺癌(nonsmall cell lung cancer, NSCLC)手术的患者中，有2.6%-7.9%的患者具有sMPLC ^[2-6]^。在普通人群中sMPLC发病率0.2%-8%(尸解研究发现为3.5%-14%) ^[7]^。对于MPLC和肿瘤肺内转移的鉴别有难度，但具有重要意义(表 1)。根据Martini和Melamed标准，sMPLC指两个肿瘤孤立，病理类型可能相同或不同。在病理相同的情况下，肿瘤一般分布在不同肺段、肺叶或不同侧肺，肿瘤原位起源且没有肺外转移或淋巴扩散的证据^[1, 8, 9]^。近年来有学者提出使用组织形态学分析或分子检测验证MPLC^[1, 10, 11]^。p53突变分析在35%-66%的sMPLC^[12, 13]^和mMPLC^[12, 14]^患者中被证实有效，已经成为一种可信的诊断工具。EGFR和KRS基因突变是肺腺癌发生的早期事件^[15, 16]^，KRAS的3p删除突变和p53变异被认为是诊断多发肿瘤的基因标记^[17]^。杂合性丢失(loss of heterozygosity, LOH)分析通过比较肿瘤组织和正常组织的单核苷酸多态性或微卫星基因型，可以区分sMPLC、mMPLC和肿瘤肺内转移^[18]^。结合LOH分析和p53突变可以提高诊断原发性肺癌的敏感性和特异性^[19]^。通过该检测发现sMPLC和mMPLC的基因谱表达存在显著差异^[18, 20]^。X染色体基因的失活发生在胚胎发展的早期，有研究用来进行女性的肿瘤检测^[21]^。阵列式基因体杂交比较法通过比较基因组拷贝数变化，也可进行sMPLC和mMPLC的鉴别诊断。值得注意的是，通过检测已经发现多个肿瘤相关基因*EGFR*、*KRAS*、*ERBB2*等均存在着肿瘤内异质性^[22-25]^，这些可能与肿瘤对治疗的反应差异有关。

**1:** MPLC的诊断标准

Martini and Melamed标准^[1]^
同时性多原发肺癌
A:肿瘤孤立且分离
B:病理类型：
1.不一致
2.相同，但在不同肺段、肺叶或不同侧
a.原位起源肿瘤
b.肿瘤之间无相同淋巴引流
c.诊断时无肺外转移
异时性多原发肺癌
A.病理不一致
B.如病理一致
1.两次肿瘤发现时间间隔≥ 2年
2.肿瘤原位起源
3.第二个肿瘤在不同肺叶或不同侧肺，但
a.没有相同淋巴回流区域的肿瘤
b.诊断时无肺外转移
Antakli修改版标准^[8]^
A.不同的病理
B.病理相同，但符合以下两条或以上情况：
1.解剖孤立
2.相关性癌前病变
3.无系统性转移
4.无纵隔内转移
5.不同的DNA倍体数
美国胸科医生学会(ACCP)指南(2007版)^[26]^
A.相同病理，但解剖位置不同
1.肿瘤位于不同肺叶
2.无N2，N3
3.无系统性转移
B.相同病理，发病时间不同
1.两次肿瘤发现时间≥4年
2.两次肿瘤均无系统性转移
C.不同病理
1.不同病理类型
2.不同分子基因特征
3.原位分别起源于肿瘤病灶

## 2 治疗

对于多原发肺癌的治疗目前仍是挑战。正如该文章作者提到，针对后发肿瘤的分期适合和患者心肺功能状况许可的前提下，手术可以作为一个选择。根据最新的美国胸科医师协会(American College of Chest Physicians, ACCP)治疗指南^[27]^，sMPLC和mMPLC均考虑行根治性手术切除，可行有创性纵隔活检、胸外影像学检查(1B级推荐)。在sMPLC患者中，两个或多个肿瘤应该分别进行分期评估，作为独立肿瘤处理^[28]^。在疑似或证实肺癌的同时同侧不同肺叶发现结节，需排除良性病变或sMPLC(1C级推荐)。在疑似或证实多发肺癌患者，推荐尽量行根治性治疗(2C级)。

关于手术的方式目前仍无统一标准，以mMPLC为例，有学者认为针对第二个肿瘤应首选单肺叶乃至双肺叶切除^[29-36]^，其次才考虑肺段或楔形切除。但也有研究将局部切除作为主要的手术方式^[8, 37-41]^。尽管肺叶局部切除与复发率增高有一定相关性^[19, 42-44]^，其复发率高于与肺叶切除^[45]^，但对于肺功能不佳的患者，目前仍然是可接受的治疗方案，因其总生存率并无明显不同，而局部切除能最大限度的保留患者的肺功能，在治疗原发肿瘤已经行肺叶切除的情况下，这样的举措对于改善患者生活质量，降低术后并发症及死亡率具有意义。对于sMPLC，手术选择包括单肺叶、双肺叶乃至一侧全肺切除^[4, 5, 29, 30, 35, 36, 46]^。如同郭连奎等人总结，解剖性切除第一个肿瘤，再对其他肿瘤进行亚肺叶切除是针对sMPLC(尤其指双侧病变)的一种安全有效的治疗手段^[4, 5, 29-31, 34, 35]^。初始手术一般选择在最大肿瘤一侧进行，随后再进行对侧手术。一项研究表明双侧使用胸腔镜手术治疗sMPLC是可行的，3年无疾病生存率77.9%^[2]^。

值得注意的是，立体定向放射治疗(stereotactic body radiation therapy, SBRT)也成为肺癌的治疗手段之一。有研究^[47-50]^显示，对于早期NSCLC，SBRT和手术治疗效果相似。目前被当做对于可切除肿瘤但不适合进行手术的患者的标准治疗方案^[51]^。回顾性研究显示SBRT治疗后患者5年存活率为51%-70%^[47, 52]^。总体存活率、复发率等方面比较，SBRT与手术无明显不同，中位存活率、无进展存活率与手术组也类似^[51, 53-55]^。

近年来，有学者针对*EGFR*和*KRAS*基因突变的患者，采用了结合手术和靶向药物治疗双侧sMPLC。在吉非替尼治疗后比较肿块变化，手术切除对吉非替尼不敏感的肿块，并继续使用吉非替尼治疗，也取得了不错的疗效^[56]^。

## 3 预后

目前研究表明mMPLC术后5年存活率20%-65%，第二个肿瘤的分期较早(Ⅰ期或Ⅱ期)往往预后较好^[29, 33, 39, 46]^。第一次手术间隔时间(< 2年)^[29, 34]^、高龄^[33]^、肿瘤切除不彻底^[29]^可预测术后存活率不高，而肿瘤不同病理类型^[57, 58]^，亚肺叶切除^[34, 49]^对5年存活率无显著影响。多发肿瘤间隔较短(< 2年)或sMPLC患者存活率比肿瘤间隔较长(≥2年)患者低^[29, 34, 35,37]^。

目前临床常用的TNM分期标准对于sMPLC和mMPLC并不合适。同一肺叶同时性卫星结节被分期为T4(Ⅲb期)，而分布在不同肺叶被认为是转移性肿瘤(Ⅳ期) ^[59]^。按照治疗标准，这些患者需要进行化疗或放疗，而非手术。但研究^[60, 61]^显示MPLC患者的生存预后明显好于Ⅲb期和Ⅳ期单肺肿瘤。事实上T4卫星肺癌患者经过根治手术后期存活率与Ib和IIa期患者相似，远高于T4侵袭性肿瘤患者^[62]^。因此，传统的TNM分期不适用与MPLC患者的治疗策略选择和预后判断。目前认为，淋巴结侵犯是预测MPLC患者预后较好的指标^[4, 63, 64]^。近期回顾性研究发现，sMPLC的预后与肿瘤大小、分化以及淋巴结转移有关，与TNM分期无关。肿瘤直径 < 0.8 cm的患者5年存活率达到100%^[65]^。

与ACCP 2007版指南相似，NCCN指南指出，MPLC患者随访需每6个月-12个月进行一次，包括病史采集、体格检查和胸部CT，一年以后改为每年胸部CT平扫一次(推荐级别2B)^[66]^。美国临床肿瘤协会推荐前2年每三个月复查一次，第3年-5年每6个月检查一次，之后每年检查一次。仅仅对于有症状的患者才需要胸部CT检查^[67]^。

## 4 总结

韩连奎等人关于MPLC的外科治疗经验总结具有重要的临床价值，随着检出率的上升，MPLC的临床治疗也正在得到越来越多的关注。区分MPLC与肿瘤肺内转移对于疾病的分期、管理和患者治疗及预后的判断具有重要意义。常规的病理形态学检查往往不足以解决诊断上的困难，辅助基因及分子生物学的检测能为诊断提供更多的信息。针对部分适合手术的患者，手术可能改善长期存活。再发肿瘤的分期早、无淋巴结转移、以及与初发肿瘤的间隔时间长(> 2年)的患者预后较好。
